# Development of a Highly Sensitive Hybrid LC/MS Assay for the Quantitative Measurement of CTLA-4 in Human T Cells

**DOI:** 10.3390/molecules28083311

**Published:** 2023-04-08

**Authors:** Dong Wei, Kristin L. Horton, John Chen, Linlin Dong, Susan Chen, Kojo Abdul-Hadi, Ting Ting Zhang, Cierra N. Casson, Michael Shaw, Tsubasa Shiraishi, Brandon Wilkinson, Chengjie Ji, Mark G. Qian

**Affiliations:** 1 Takeda Pharmaceutical Company International Co., 35 Landsdowne Street, Cambridge, MA 02139, USA; 2 NovaBioAssays LLC, 52 Dragon Ct, Suite 3B, Woburn, MA 01801, USA

**Keywords:** T-lymphocyte-associated protein 4 (CTLA-4), human T cells, LC/MS, quantification

## Abstract

Cytotoxic T-lymphocyte-associated protein 4 (CTLA-4) is a check point protein expressed on the surface of T cells and plays a central role in regulating the immune response. In recent years, CTLA-4 has become a popular target for cancer immunotherapy in which blocking CTLA-4 can restore T-cell function and enhance the immune response against cancer. Currently, there are many CTLA-4 inhibitors in a variety of modalities, including cell therapies, which are being developed in both preclinical and clinical stages to further harness the potential of the target for the treatment of certain types of cancer. In drug discovery research, measuring the level of CTLA-4 in T cells is important for drug discovery and development because it provides key information for quantitative assessment of the pharmacodynamics, efficacy, and safety of the CTLA-4-based therapies. However, to our best knowledge, there is still no report of a sensitive, specific, accurate, and reliable assay for CTLA-4 measurement. In this work, an LC/MS-based method was developed to measure CTLA-4 in human T cells. The assay demonstrated high specificity with an LLOQ of 5 copies of CTLA-4 per cell when using 2.5 million T cells for analysis. As shown in the work, the assay was successfully used to measure CTLA-4 levels in subtype T-cell samples from individual healthy subjects. The assay could be applied in supporting the studies of CTLA-4-based cancer therapies.

## 1. Introduction

The adapted immune response of T cells is determined by T-cell antigen-receptor (TCR) signaling, as well as the co-stimulatory and co-inhibitory molecules such as CD28 and cytotoxic T-lymphocyte-associated protein 4 (CTLA-4). The carefully regulated dual CD28/CTLA-4 mechanisms ensure that the anti-pathogen and anti-tumor immune effects are carried out without autoimmune responses and collateral damage to normal tissues [1]. The expression of CTLA-4 has been demonstrated in lymphoid and myeloid cells as well as tumor cells. It was proposed that the blockage mechanism of CTLA-4 could be used in cancer treatments. The remarkable clinical results of ipilimumab, a CTLA-4 blocker, and its subsequent approval for the treatment of melanoma, opened a new era in immune-oncology [2,3,4]. While the checkpoint blockers that include anti-CTLA-4 and anti-programmed cell death protein-1/ligand-1 (PD1/PDL1) have demonstrated unprecedented efficacy in treating a number of cancers such as non-small cell lung cancer, melanoma, and urothelial carcinoma, the response rate remains unsatisfactory [2]. In contrast to CD28, the biology of CTLA-4, as well as the mechanism of action of anti-CTLA4 therapies, is not fully understood. While CD28 is constitutively expressed in all T cells, CTLA-4 is constitutively expressed only in regulatory T cells. Its level is low or not detectable in naïve, resting T cells but can be measured in induced T cells, as well as CD4 and CD8 T cells in cancer [5]. In addition, it was found that most CTLA-4 resides in the cytoplasmic vesicles, which could be trafficked to the plasma membrane upon activation.

The expression of CTLA-4 in cells has been evaluated by qPCR at mRNA levels, as well as by flow cytometry, Western blot, and immunohistochemistry including quantitative digital image analysis [5,6]. While these methodologies have been used extensively in the research of CTLA-4, they possess some inherent limitations including but not limited to insufficient sensitivity, semi-quantification, and/or the inability to differentiate and measure the intracellular proteins. Thus, a more sophisticated quantitative assay for CTLA-4 in human immune cells is needed which, if available, will enable better understanding of CTLA-4 biology, including the protein’s baseline expression, turnover, and mechanism of T-cell activation. Equally important though, is the reliable measurement of CTLA-4 levels which would allow for a detailed quantitative pharmacological and toxicological assessment of the target in cancer immunotherapies. For example, the measured level can be used to determine the threshold of CTLA4 copies and/or the ratio of CD28 to CTLA4 required for the activation of CD8+ cytotoxic T cells for subsequent tumor killings. In addition, knowledge of the CTLA-4 level in diverse biological matrices and settings may also facilitate immune oncology drug discovery, including the understanding of target engagement, patient selection and stratification, prognostic and predicative assessment of drug responses. For these important reasons, we developed a quantitative method to measure the CTLA-4 level in human T cells using the hybrid liquid chromatography–mass spectrometry (LC/MS) technology.

In recent years, the LC/MS-based methodology, in particular when coupled with immunoprecipitation, has been widely used for the analysis of a broad spectrum of protein therapeutics as well as biomarkers [7,8,9]. For example, a hybrid LC/MS method has been developed to measure PD-1 and PD-L2 in human tumor samples using a proteomics approach as well as a hybrid LC/MS method [10,11,12].

In this paper, we describe the development of a highly specific and sensitive hybrid LC/MS assay that quantitatively measures the CTLA-4 level in human immune cells with a low detection limit of five copies per cell. The method was used to assess the CTLA-4 level from various T- and B cells isolated from human blood samples. As shown in the paper, the method could potentially be used as a tool for the study of CTLA-4 biology and the measurement of CTLA-4 biomarkers in patient blood samples.

## 2. Results and Discussion

### 2.1. Assay Design

**Figure 1:**
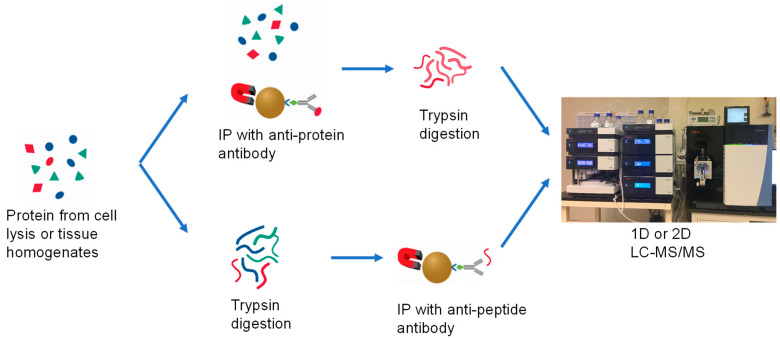
Two typical workflows for immunoprecipitation of the proteins from biological samples.

Based on previous publications, the CTLA-4 protein is localized in the intracellular compartments as well as on the plasma membrane. Our assay is thus designed to allow for extraction and quantitative measurement of the total CTLA-4 level from the cell samples. There are two approaches in the immune extraction of the proteins from biological samples in the hybrid LC/MS methodologies shown in Figure 1. In the first approach, the target protein of interest is selectively captured by an antibody conjugated on magnetic beads. The captured protein is then enzymatically digested on or off beads into peptides. One or more unique peptides from the protein digest, from those with the highest mass-spectrometric response and the lowest interference from matrices, are typically chosen as surrogates to measure the protein abundance in the samples.

The second approach is termed Stable-Isotope Standards and Capture by Anti-Peptide Antibodies (SISCAPA), in which the total protein is extracted from the samples and enzymatically digested into peptides. An antibody against a targeted surrogate peptide is conjugated onto magnetic beads or packed in a column and is used to selectively extract the target surrogate peptide for analysis by LC/MS.

Since CTLA-4 is a cross-membrane protein with hydrophobic domains which may lead to loss due to aggregation and/or non-specific absorption onto vial surfaces, the second approach with the anti-peptide antibody is thus believed to be superior and was therefore used for the work described subsequently in the report. In our study, the total protein from the cell samples was dissolved directly in 8 M urea, denatured, alkylated, and digested into peptides. This approach minimized the loss of the CTLA-4 analyte during the sample–preparation process and thereby maximized its recovery.

The assay was designed to measure the CTLA-4 protein in human T cells, which are typically isolated from human whole-blood samples and thus are inherently of limited sample quantities especially for cancer patients. To achieve the best sensitivity for such samples, a 2D nano-LC/MS system was employed. The extracted samples were first injected into a trap cartridge to enrich the analyte. Then, the analyte was eluted into a nano-LC column running at a nano flow prior to MS detection.

### 2.2. Selection of the Reference Standard and Surrogate Peptide

Human CTLA-4 is a homodimer membrane protein that includes an extracellular domain (ECD 36–161, UniProt P16410), a hydrophobic transmembrane section (162–182), and a cytoplasmic domain (183–223). For practical reasons, recombinant proteins that include the extracellular binding domain (36–161) were used as the reference material for method development and preparation of spiked standard/QC samples.

One of the key steps in developing a hybrid LC/MS assay is to choose a surrogate peptide that could render the optimal sensitivity, specificity, and minimal interference from biological matrices. In the hybrid method that uses an anti-peptide antibody to selectively extract the analyte (SISCAPA), the surrogate peptide is used for dual purposes: as the target antigen to generate polyclonal antibodies for immunocapture, and as the surrogate analyte for the target protein in LC/MS quantitation. Therefore, the surrogate peptide selected should have a high immunogenic potential after anchoring to a carrier protein for inducing polyclonal antibodies in rabbits. In our method development, the recombinant CTLA-4 was digested and tuned on the Thermo triple–quadrupole mass spectrometer. The most sensitive transition obtained was from peptide GIASFV[C]EYASPGK, a y8+ ion at *m*/*z* 911.4 from the doubly charged parent ion at *m*/*z* 743.3. Furthermore, the peptide was chosen as the surrogate peptide for its additional favorable attributes: (1) a unique peptide based on protein blast in human–protein databases; (2) a relative long peptide which was hypothesized to be favorable in polyclonal antibody generation.

### 2.3. Assay Performance

**Figure 2:**
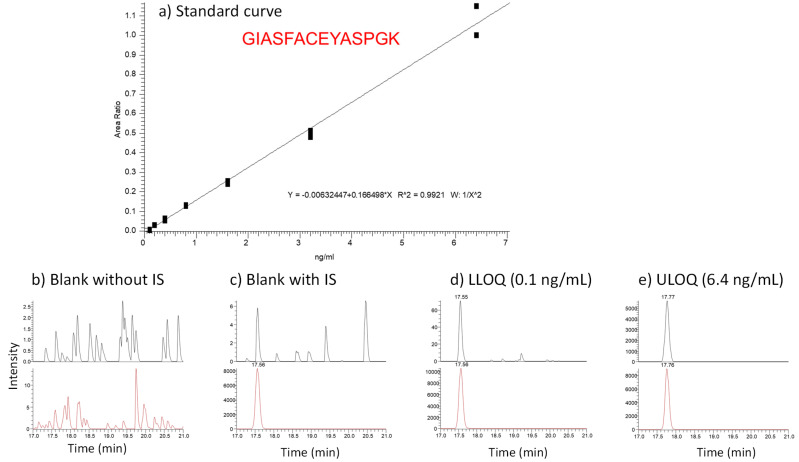
The standard curve and representative LC-MRM MS chromatograms.

A recombinant CTLA-4 protein with Fc was used as the reference material and spiked into a surrogate matrix (0.1% BSA in PBS) to prepare standard curves. A representative calibration curve and related selected extracted ion chromatograms are shown in Figure 2. The linearity of the standard curve was R^2^ = 0.9921. The accuracy of the back-calculated standard samples was within ±20% of nominal concentrations. The dynamic range of the assay was 0.1 to 6.4 ng/mL with the lower limit of quantification (LLOQ) at 0.1 ng/mL. (Table 1).

**Figure 3:**
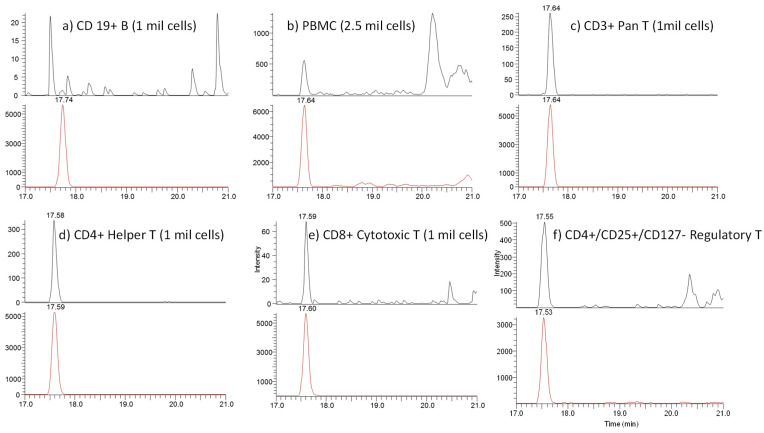
The representative LC-MRM MS Chromatograms for cell samples with analyte (**top**) and IS (**bottom**).

The assay also showed high specificity. In the chromatogram of the blank samples in the surrogate matrix, no interference peaks were found (Figure 2). Among all the samples tested, the CTLA-4 level in the CD19+ B cell samples was below the LLOQ (Figure 3).

To determine the total percentage recovery of the reference standard spiked in human cell samples compared to that spiked in the surrogate matrix (0.1% BSA in PBS), a recovery experiment was carried out by spiking the reference standard at 0.400 ng/mL into B-cell and pan T-cell samples (0.5 million cells for each). The samples were measured using a standard curve in a surrogate matrix. The recovery was found to be 106% in spiked B-cell samples, which have no measurable level of endogenous CTLA-4. The recovery was slightly higher in the spiked pan T-cell samples (140%), which however included a measurable level of endogenous CTLA-4 at 0.253 ng/mL in the 0.5-million-cell sample. Since the endogenous CTLA-4 protein was different from the spiked reference standard, determining the true recovery of the analyte during the extraction and tryptic digestion was not feasible. However, the data demonstrated a comparable recovery of the CTLA-4 reference protein spiked in a surrogate matrix versus that spiked in B- and T-cell samples (Table 2).

To determine the parallelism of the assay method for the T-cell samples, the same batch of CD3+ pan T cells was used to prepare samples at 0.2, 0.5 and 1 million cells per well. The samples were measured against a standard curve. The measured CTLA-4 levels from cell counts in the range of 0.2 to 1 million cells per well showed acceptable parallelism results with %CV at 11.4%. (Table 2)

### 2.4. Measurement of Human Blood Cell Samples

Human cells from multiple donors were purchased from commercial sources and measured for the CTLA-4 levels. The representative extracted ion chromatograms from the samples are shown in Figure 2 and the results are listed in Table 3. The copy number of CTLA-4 protein per cell was calculated based on the measured total CTLA-4 concentration in pg/mL using the following equation with the assumption that each endogenous CTLA-4 protein is a homodimer with two copies of the surrogate peptide which is the same as the reference standard.

(1) $$\left[CTLA-4\right]\left(\frac{copy}{cell}\right)=\frac{\left[CTLA-4\right]\left(\frac{pg}{mL}\right)\times \left[Final\;Sample\;Volume\right]\left(mL\right)\times 6.022\times 10^{23}}{\left[Number\;of\;Cells\;in\;Sample\right]\times 10^{12}\left(\frac{pg}{g}\right)\times 115,000\left(MW\;of\;CTLA-4\;Standard\right)}$$

It was found that the CD4+/CD25+/CD127-regulatory T cells showed the highest CTLA-4 levels, averaging between 349 and 1132 copies per cell in the samples from two donors. In the CD19+ B cell samples, the CTLA-4 level proved to be below the LLOQ (equivalent to < five copies per cell). The levels of CTLA-4 from other cell types were: PMBC 46–98 copies per cell; CD3+ pan T 67–112 copies per cell; CD4+ Helper T 27–77 copies per cell; and CD8+ cytotoxic T <12.5–19 copies per cell. The results are consistent with previous reports. It should be pointed out that since regulatory T cells have significantly higher levels of CTLA-4 compared with other type of cells, they could elevate the CTLA-4 level of the cell samples that contain them, such as PBMCs and CD3+ Pan T cells. This seemed to be the case as the CTLA-4 levels of these two types of cell showed higher copies per cell vs. the CD4+ helper T and CD8+ cytotoxic T cells. The result of the study also indicates that the purity of the cell samples is important since a small impurity of regulatory T cells could artificially elevate the overall measured CTLA-4 level in other types of cell samples, such as the helper T and cytotoxic T samples. The purity of most of the cells was > 90%. Among the different T-cell types, the CD8+ cytotoxic T cells demonstrated the lowest CTLA-4 level and the three samples measured were with results close to the LLOQ of 12.5 copies per cell.

PD1/PD-L1 and CTLA-4 represented the two central classes of targets in immune oncology that demonstrated promising efficacies after inhibition. PD-L1 was found elevated on the cell surface of certain tumors such as non-small cell lung cancer. PD-L1 immunohistochemistry (ICH) assays were approved by the FDA as a companion diagnosis for risk/benefit predictions [13]. LC/MS assays were also developed to quantitatively measure PD1/PD-L2 in tumor tissues [10,11,12].

The expression of CTLA-4 was reported to be predominantly in T cells, as well as other blood cells and tumor cells. The research into CTLA-4 biology has been focused on its function in T cells, especially the immune suppression of regulatory T cells [14]. Quantitative imaging analysis by CTLA-4 immunohistochemistry (ICH) was used to measure the CTLA-4 expression of the tumor-infiltrating lymphocytes [5]. Previous studies have shown that CTLA-4 is predominantly and constitutively expressed in T_Reg_ cells [15], and the CTLA-4 expression is higher in CD4+ T cells than in CD8+ T cells from healthy subjects [16]. In this work, an LC/MS method was developed and used to quantitatively measure the total of CTLA-4 in subtypes of T cells from healthy subjects. The results showed that the CTLA-4 levels in human T cells are T_Reg_ >> CD4+ T > CD8+ T, which are consistent with the abovementioned publications. The results also demonstrated significant variations (two-three-fold) among subjects in each T-cell subtype. The LC/MS assay demonstrated the feasibility of quantitatively measuring CTLA-4 in fewer than one million subtype T cells, thus enabling its potential application as a companion diagnosis together with liquid biopsy in immunotherapy development. There have been other publications that reported the measured soluble CTLA-4 levels in biological fluids. However, the identities of the analytes were subject to debate [17,18]. The LC/MS assay developed herein has the potential to measure soluble CTLA-4 ECD in biological fluid and tissue samples with high specificity and less ambiguity.

## 3. Materials and Methods

### 3.1. Chemicals and Reagents

Recombinant human CTLA-4 extracellular domain (ECD) protein with a His-tag was manufactured at Wuxi Biologics (Shanghai, China). Recombinant human CTLA-4 Fc Chimera Avi-tag Protein (Biotin-labeled) was obtained from R&D Systems (Minneapolis, MN, USA). The surrogate tryptic peptide of CTLA-4 H2N-GIASFVC(CAM)EYASPGK^-OH (Cys modified by iodoacetamide with a carbamidomethyl group) and its stable isotope-labeled internal standard H2N-GIASFVC(CAM)EYASPGK^-OH (K +8 Da) were purchased from Thermo Scientific (Waltham, MA, USA). The Sulfo ChromaLink Biotin was obtained from Trilink Biotech (San Diego, CA, USA). The Dynabeads™ MyOne™ Streptavidin T1 was purchased from Thermo Fisher (Chicago, IL, USA). Acetonitrile, water, and formic acid (FA) were of HPLC grade or higher. A mass spectrometry grade Trypsin/Lys-C Rapid Digestion kit was purchased from Promega (Madison, WI, USA). The Tris buffer (0.1 M, pH 7.5), 1X PBS buffer (pH 7.4), PBST1 (PBS with 0.01% Tween 20), PBST5 (PBS with 0.05% Tween 20), 10 M urea, 100 mM DTT in 1 M pH 7.5 Tris-HCl, 240 mM iodoacetamide (IAA) in 100 mM pH 7.5 Tris-HCl, 3 M sodium acetate (pH 5.2), 4% BSA-PBST, 0.05% CHAPS in 1X PBS, 25 mM HCl in water, were prepared in-house.

### 3.2. Cell Samples

Human blood cells (B, CD4+ and CD8+ T, regulatory T) were purchased from Hemacare (Los Angeles, CA, USA) and stored at −80 °C until use. The cell count number was provided by the vendor. The Jurkat T-cell line stably expressing human CTLA-4 and an IL2-Luciferase reporter was purchased from Promega (Cat# CS186903, Madison, MI, USA) and cultured in the following media: Gibco RPMI 1640 90% (Thermo Fisher Scientific, Waltham, MA, USA); HyClone FBS 10% (Fisher Scientific, Hampton, NH, USA); MEM non-essential amino acids 1X; sodium pyruvate 100 mM 1X; hygromycin B 200 ug/mL (Thermo Fisher Scientific); and Promega G418 (Fisher Scientific). The cells were maintained in a culture medium between 2 × 10^5^ and 2 × 10^6^ cells/mL. Cells were passaged by diluting with a fresh culture medium to a seeding density of 4 × 10^5^ cell/mL (2-day passage) or 2–3 × 10^5^ cells/mL (3-day passage). The cells were counted with a Cellometer (Nexcelom, Lawrence, MA, USA), washed with PBS (×2), centrifuged, and the harvested pellet was stored at −80 °C until use.

Human T and B cells in 1 mL CryoStor freezing media were purchased from Hemacare, with each vial containing 25 million Cryo-PBMCs, 10 million CD3+ Pan T cells, 25 million PB Cryo-CD4 cells, 10 million PB Cryo-CD8 cells, 10 million PB Cryo-B cells, or 1 million PB Cryo-CD127D T_Reg_ cells.

### 3.3. Generation of Anti-Peptide Polyclonal Antibody

An anti-peptide polyclonal antibody was generated at BBI Solutions (Portland, ME, USA). The peptide was extended by a Cys at the n-terminal and conjugated with keyhole limpet hemocyanin (KLH). A total of 3 rabbits were used for immunization and antisera collections. The primary immunization was administered with 250 µg of the antigen with Complete Freund’s adjuvant. Subsequent boosts consisted of 125 µg of the antigen with Incomplete Freund’s adjuvant every 3 weeks. The rabbits were bled and tested for titers. The purifications were carried out 2.5 and 4 months after the initial challenges. The polyclonal antibody was purified with Protein A and peptide antigen-affinity chromatography.

### 3.4. Preparation of Calibration Standard and Internal Standard

Calibration standards at 0.1, 0.2, 0.4, 0.8, 1.6, 3.2, and 6.4 ng/mL were prepared in 0.1% BSA in PBS solution (surrogate matrix) by dilution from a stock solution of recombinant human CTLA-4 Fc chimera Avi-tag protein at 1.5 mg/mL. The internal standard solution of 1 nM in 5% acetonitrile in water was prepared by diluting the stable labeled internal standard stock solution that was prepared by dissolving the peptide powder in 5% acetonitrile in water.

### 3.5. Sample Processing

Cell sample denaturation and homogenization: A volume of 0.1 mL of cell mixtures was aliquoted into a vial containing 98 mg of urea which led to the final mixture volume of about 0.2 mL with the urea concentration at 8 M, adequate for protein denaturing. The suspended cells were then homogenized using an ultrasonics processor with a tip probe (Zhengzhou TCH Instrument Co., Zhengzhou, China), followed by mixing with 20 μL of 1 nM internal standard solution.

Calibration standards denaturation: An aliquot of 25 µL of each calibration standard was transferred to a 96-well Thermo Kingfisher plate containing 28 mg urea per sample well and mixed with 20 μL of 1 nM internal standard solution.

Tryptic digestion: Each of the denatured cell samples and the calibration standards was mixed with 10 µL of 50 mM DTT in 1 M pH 7.5 Tris-HCl. The plate was then incubated with shaking at 550 rpm at 37 °C for 1 h. An aliquot of 10 µL of 120 mM IAA in 100 mM pH 7.5 Tris-HCl was added to each well and the samples were incubated in the dark with shaking at 550 rpm at room temperature for 30 min, then under light for 20 min. Each sample was mixed with 500 µL of 100 mM pH 7.5 Tris-HCl and 50 µL of 0.1 µg/µL of Trypsin/LysC in 100 mM pH 7.5 Tris-HCl. The samples were then digested overnight by incubating with shaking at 550 rpm at 37 °C. To adjust the pH, 20 µL 3 M sodium acetate (pH 5.2) was added to each well after digestion.

Immunoaffinity enrichment: The anti-peptide antibody was biotinylated with the Sulfo ChromaLink Biotin following the vendor’s protocol. An aliquot of 20 µL of 0.075 mg/mL of biotinylated anti-peptide antibody was added to each well containing the digested sample. The plate was incubated at 8 °C with shaking at 850 rpm for 90 min. Each sample was then mixed with 10 µL Streptavidin T1 Dynabeads and incubated at 25 °C for 1 h at 950 rpm, and then placed onto a Kingfisher system. All samples were subjected to duplicate washes with 0.05% CHAPS in 1X PBS (400 µL), a single wash with 0.017% CHAPS in 1X PBS (400 µL) prior to eluting with 150 µL of 25 mM HCl. The eluted peptides were injected for LC-MS analysis.

### 3.6. Liquid Chromatography and Mass Spectrometry

A Thermo Ultimate 3000 UHPLC system and Ultimate 3000 RSLC nano system (Waltham, MA, USA) interfaced to a Thermo TSQ Quantiva triple-quadrupole mass spectrometer with an EasySpray nano source (Waltham, MA, USA) were used for peptide quantitation. A total of 100 µL of immunoaffinity-enriched peptide eluates from each sample was injected onto a C18 PepMap trap column (0.3 mm × 5 mm, 5 µm, 300 Å, Thermo Fisher) with 0.5% TFA in water at the flow rate of 0.2 mL/min for 3 min. Subsequently, the peptides eluted from the C18 trap column were separated using a PepMap^TM^ C18 Column (3 μm, 100 Å, 75 μm × 15 cm, Thermo Fisher) running at 60 °C. Mobile phases A and B were 0.1% FA in water, and acetonitrile containing 0.1% FA, respectively, and the total flow was 0.6 µL/min.

The nano-LC gradient employed was as follows: maintained at 3% B for 6 min for loading; then ramped to 11% B in one minute; continued to ramp to 19% B in 15 min; and then held at 80% B for 2 min. The mass spectrometer was operated at an MRM positive ionization mode, and the transitions at 743.3 > 911.4 and 747.3 > 919.4 were used for the analyte and corresponding internal standard, respectively.

The LC/MS data were acquired and processed using Thermo Xcaliber software 4.0. Linear calibration curves were established based on the peak area ratio (analyte/IS) vs. concentration using 1/X weighting to enable the assay optimization as well as quantification of unknowns.

## 4. Conclusions

A highly sensitive and specific hybrid LC/MS assay has been developed and applied to quantitatively measure CTLA-4 levels in human blood cells. The assay is sensitive enough to measure as low as 5 copies of CTLA-4 per cell using 2.5 million cells per sample, or 12.5 copies per cell using 1 million cells per sample. The measurement was believed to be reliable based on the surrogate spiked CTLA-4 recovery assessment in relevant cell matrices. The novel method provided a practical quantitative method for the total of CTLA-4 level in human T cells, which could be widely applicable in studying CTLA-4 as an important drug target for developing immunotherapies for cancer treatment.

## Figures and Tables

**Table 1:** A Representative Standard Curve of Total CTLA-4 Assay.

Sample Type	Nominal CTLA-4 Conc. (ng/mL)	Back Calculated Conc. (ng/mL)	%Diff
Std1-1	0.1	0.07	−30% *
Std1-2	0.1	0.092	−8%
Std2-1	0.2	0.23	15%
Std2-2	0.2	0.131	−34% *
Std3-1	0.4	0.379	−5%
Std3-2	0.4	0.427	7%
Std4-1	0.8	0.84	5%
Std4-2	0.8	0.815	2%
Std5-1	1.6	1.581	−1%
Std5-2	1.6	1.489	−7%
Std6-1	3.2	3.122	−2%
Std6-2	3.2	2.94	−8%
Std7-1	6.4	6.954	9%
Std7-2	6.4	6.059	−5%

* Excluded from regression.

**Table 2:** Spiked Recovery and Parallelism of the CTLA4 Assay.

Sample Type	Cell Number (Million)	Nominal Conc. (ng/mL)	Back Calculated Conc. (ng/mL)	%Diff	
Std 1		0.1	0.081	−19%	
Std 2		0.2	0.199	0%	
Std 3		0.4	0.477	19%	
Std 4		0.8	0.822	3%	
Std 5		1.6	1.60	0%	
Std 6		3.2	3.12	−3%	
		**Spike Conc. (ng/mL)**	**Measured Conc. (ng/mL)**	**Accuracy (%)**	**Response Factor**
Human B Cell	0.5	0	0.068 (BLPQ)	N/A	
Human B Cell	0.5	0.800	0.920	106	
Human T Cell	0.2	0	0.118	N/A	0.591
Human T Cell	0.5	0	0.253	N/A	0.506
Human T Cell	1.0	0	0.635	N/A	0.635
Human T Cell	0.5	0	1.474	140	
CV of Response Factors		0.8			11%

**Table 3:** Measured Total CTLA-4 Concentration in Human T, B, and Cultured Jurkat Cells.

Cell Samples	Lots	Purity (Viability)	Gender	Age	Measured CTLA-4 (Copy per Cell)
CD19+ B	14034019	>90% (>95%)	M	29	<5
	16039855	N/A (>95%)	M	23	46
PBMC	20062721	N/A (>95%)	F	51	98
	20062803	N/A (>95%)	F	43	46
	20061999	>90% (>95%)	M	39	67
CD3+ Pan T	20063759	>90% (>95%)	M	54	112
	16038257	>90% (>95%)	M	29	97
	20063853	>90% (>95%)	M	58	77
CD4+ Helper T	20064018	>90% (>95%)	M	38	27
	21067658	>90% (>95%)	M	27	56
	18048894	>90% (>95%)	M	52	19
CD8+ Cytotoxic T	18048997	>90% (>95%)	F	39	13
	19057442	>90% (>95%)	M	23	<12.5
CD4+/CD25+/CD127-Regulatory T	20062260	>80% (>95%)	M	47	1132
	20063080	>80% (>95%)	M	42	349
CTLA-4 Expressing Jurkat T cell					23,000

## Data Availability

The data presented in this study are available up on request.

## Sample Availability

Samples in the study are not available from the authors.
